# A CD64/FcγRI-mediated mechanism hijacks PD-1 from PD-L1/2 interaction and enhances anti-PD-1 functional recovery of exhausted T cells

**DOI:** 10.3389/fimmu.2023.1213375

**Published:** 2023-08-09

**Authors:** Victor Joo, Constantinos Petrovas, Laurence de Leval, Alessandra Noto, Michel Obeid, Craig Fenwick, Giuseppe Pantaleo

**Affiliations:** ^1^ Service of Immunology and Allergy, Department of Medicine, Lausanne University Hospital, University of Lausanne, Lausanne, Switzerland; ^2^ Institute of Pathology, Lausanne University Hospital, University of Lausanne, Lausanne, Switzerland; ^3^ Lausanne Center for Immuno-oncology Toxicities (LCIT), Service of Immunology and Allergy, Department of Medicine, Lausanne University Hospital, University of Lausanne, Lausanne, Switzerland; ^4^ Swiss Vaccine Research Institute, Lausanne University Hospital, University of Lausanne, Lausanne, Switzerland

**Keywords:** PD1 (programmed cell death protein 1), immunotherapy, internalization, FcγRI(CD64), T cell, mAb, downregulation

## Abstract

Therapeutic monoclonal antibodies (mAb) targeting the immune checkpoint inhibitor programmed cell death protein 1 (PD-1) have achieved considerable clinical success in anti-cancer therapy through relieving T cell exhaustion. Blockade of PD-1 interaction with its ligands PD-L1 and PD-L2 is an important determinant in promoting the functional recovery of exhausted T cells. Here, we show that anti-PD-1 mAbs act through an alternative mechanism leading to the downregulation of PD-1 surface expression on memory CD4^+^ and CD8^+^ T cells. PD-1 receptor downregulation is a distinct process from receptor endocytosis and occurs in a CD14^+^ monocyte dependent manner with the CD64/Fcγ receptor I acting as the primary factor for this T cell extrinsic process. Importantly, downregulation of surface PD-1 strongly enhances antigen-specific functional recovery of exhausted PD-1^+^CD8^+^ T cells. Our study demonstrates a novel mechanism for reducing cell surface levels of PD-1 and limiting the inhibitory targeting by PD-L1/2 and thereby enhancing the efficacy of anti-PD-1 Ab in restoring T cell functionality.

## Introduction

In recent years, anti-PD-1 monoclonal antibodies have become a conventional line of immunotherapy in the successful treatment of numerous advanced cancer malignancies. Antibodies targeting the PD-1–PD-L1/2 interaction have now been approved as first-line/second-line therapies for melanoma, lymphomas, lung cancers, bladder cancer, gastroesophageal cancer, head and neck squamous cell cancer, renal cell cancer, and liver cancer (1). Despite the promising success in initial treatment responses, a large proportion of patients are unresponsive or partially responsive and can acquire primary and/or secondary resistance to PD-1 therapy while only a fraction achieve complete responses from anti-PD-1 therapy (2). While PD-1 therapy sees pervasive use in the current oncology field, T cell exhaustion through PD-1 and rescue of exhausted CD8^+^ T cells was initially recognized in chronic viral infections such as LCMV and HIV (3, 4). Thus, PD-1 therapy has also been investigated concurrently for use in the treatment of challenging infectious diseases such as HIV, HBV, HCV, tuberculosis, malaria, and SARS-CoV-2 (5–9). However, confounding factors such as the variability of virulence, context of infection status, modulation of immune populations, and unstable latency cycles obscure the efficacy of PD-1 therapies in infectious diseases (10–12). More work is needed to better understand the key T cell intrinsic and extrinsic components that promote an effective immune response to anti-PD-1 therapy.

The principal inhibitory mechanism of PD-1 is contingent on its recruitment of SHP2 and the ensuing SHP2 mediated dephosphorylation of an activated TCR complex and CD28 receptor. Specifically, anti-PD-1 antibodies have been shown to impart a restorative effect mainly through the CD28 costimulatory pathway and its downstream mediators (13–16). Numerous studies have also shown that anti-PD-1 therapies increase cytokine production and proliferative capacities in memory T cells, described as rescue or reinvigoration from T cell exhaustion (3, 14, 15, 17).

Despite these significant advances in elucidating the mechanism of action of anti-PD-1 therapy on restoring functional T cell responses, the trajectory of bound anti-PD-1 antibody-receptor complex and its effect on surface expression of PD-1 in memory T cells has yet to be fully understood. A number of studies have sought to address the spatial localization and transport of PD-1 receptor itself. The work by Meng, X. et al. (18) reported that upregulated PD-1 was internalized, ubiquitinated, and degraded by the proteasome after activation with PHA in a Jurkat cell line. In the paper by Bricogne, C. et al. (19), surface PD-1 was shown to be shed in ectosomes and regulated by TMEM16F ion channels in the presence of ionomycin treatment on PD-1-GFP expressing Jurkat cells. In addition, an early study (20) showed that 3-day OVA peptide stimulated OT-II cells were shown to have the PD-1 receptor localized in endosomal compartments proximal to Golgi matrix proteins and were co-expressed with the trans-Golgi network while absent from recycling endosomes or lysosomes. While a clear agreement on the internalization or transport route of the PD-1 receptor has not been reached, another question remains if PD-1 targeting antibodies affect internalization of the PD-1 receptor within the same transport pathways and whether any potential Fc-dependent mechanisms occur on bound anti-PD-1 antibodies.

The IgG4 subclass of commercially approved anti-PD-1 antibodies such as pembrolizumab (Keytruda^®^) and nivolumab (Opdivo^®^) is known to have minimal Fcγ receptor (FcγR) binding which abrogates antibody-mediated interactions such as antibody-dependent cell-mediated cytotoxicity (ADCC) and antibody-dependent cell-mediated phagocytosis (ADCP) in comparison to the more clinically prevalent IgG1 subclass of typical monoclonal antibody therapies. In addition, the C1q binding site is disrupted in the IgG4 C_H_2 domain BC and FG loops, resulting in the inhibition of complement dependent cytotoxicity (CDC) (21). These intrinsic, non-activating characteristics of IgG4 antibodies are currently being utilized or investigated for the blocking/neutralization of antigens without engagement of host Fc effector functions (22–24). However, it has been previously established that IgG4 antibodies are not completely safeguarded from FcγR binding and will bind under high avidity or aggregation conditions with high affinity to FcγRI (CD64) and to a lesser degree, FcγRIIA (CD32a), FcγRIIB (CD32b), and FcγRIIIA (CD16a) (25). Specifically, the high affinity Fc receptor CD64 was initially believed to be constitutively saturated with monomeric antibodies from the serum, thereby outcompeting binding with monoclonal antibodies. However, it is now known that IgG4 antibodies that are complexed or opsonized can bind CD64 with a binding affinity on the same order of magnitude as IgG1 and IgG3 antibodies (26). Inside-out-signaling has also been shown for CD64, whereby activation by local cytokines/chemokines induces ITAM phosphorylation on the γ-chain, FcγR clustering, cytoskeletal rearrangement, and conformational changes which amplifies the IgG4 binding avidity (27, 28).

The phenomenon of antibody induced surface downregulation is a type of antigenic modulation exerted by adjacent cells involving the transfer of cell surface molecules from a donor cell to an effector/acceptor cell. When antibodies bind to their target antigens on donor cells, the effector cell removes the antibody-antigen immune complexes from the surface of the donor cell, leading to the endocytosis of the antibody-antigen immune complex and/or surface expression of exchanged antigen on the effector cell (29, 30). This process can be viewed as an antibody dependent model of trans-endocytosis or trogocytosis, which is exemplified by exchange of surface receptors between T cells and APCs facilitated by the close contact of the immunological synapse in TCR and peptide-MHC interactions (31–34). In the antibody dependent model of surface downregulation, the Fc domain of antigen bound antibodies functions as the ligand for FcγR-expressing cells, enabling the transfer of antibody-bound antigen to the effector cell and loss of antigen expression in the donor cell.

Here, using primary T cells, we investigated two pharmacologic mechanisms inherent to monoclonal antibody studies: antibody internalization and surface downregulation of its target PD-1 receptor. We utilized commercial blocking anti-PD-1-IgG4 antibodies pembrolizumab and nivolumab as well as NB01, a novel non-blocking anti-PD-1-IgG4 antibody that binds to the opposite face of the PD-1 relative to the PD-L1/2 interaction site (15). We sought to investigate whether (1) anti-PD-1 antibodies functioned differently based on their binding site; (2) if the combination of blocking and non-blocking anti-PD-1 antibodies produced an additive effect; (3) whether the different anti-PD-1 antibodies were capable of downregulating surface PD-1 levels and which mechanisms were involved. Furthermore, we investigated the impact of the downregulation of surface PD-1 on the functional recovery of T cell exhaustion. Our results confirm that anti-PD-1 therapies are internalized in PD-1^+^ memory T cells at a fixed rate but the downregulation of surface PD-1 receptor is dependent on the presence of CD14^+^ HLA-DR^+^ monocytes. The frequency of monocytes in blood mononuclear cells proved to be a consistent indicator for the effect of surface PD-1 downregulation and antibody-FcγR interactions were mediated primarily through CD64. Finally, the downregulation of surface PD-1 was shown to strongly contribute to the functional recovery of PD-1^+^CD8^+^ T cells.

## Results

### Surface PD-1 downregulation observed in exhausted memory T cells from HIV infected donors when treated with anti-PD-1 antibodies

**Figure 1:**
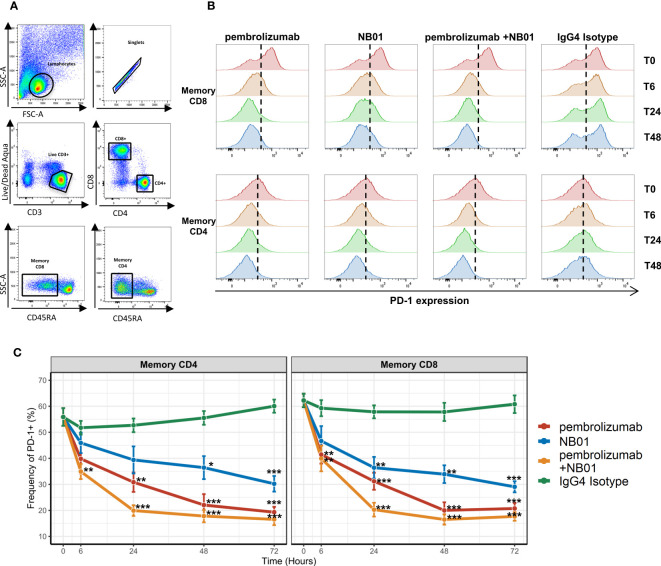
PD-1 antibody mediated downregulation of surface PD-1 receptors on memory T cells from chronic HIV donors. **(A)** Gating strategy for memory CD4 and CD8 T cells. **(B)** Representative histogram profiles of PD-1 expression on memory T cells over days when treated with 5 μg/mL of PD-1 antibodies pembrolizumab, NB01, or the combination of pembrolizumab and NB01. **(C)** Average kinetics of surface PD-1 downregulation over 72 hours with same antibody conditions as **(B)** in 5 donors. Data represented as mean ± SD. * denotes p ≤ 0.05; ** denotes p ≤ 0.01; *** denotes p ≤ 0.001 in two-tailed unpaired *t* test with Welch’s correction compared to IgG control.

In our preliminary observations, blood mononuclear cells isolated from chronically infected HIV+ donors were treated with various anti-PD-1-IgG4 antibodies (pembrolizumab, NB01, or pembrolizumab + NB01) in order to evaluate any intrinsic immunomodulatory effects on exhausted memory CD4^+^ and CD8^+^ T cells. Due to the chronic HIV antigen exposure, the donors’ memory T cells were functionally exhausted with physiologically high expression of the PD-1 receptor and changes in surface PD-1 levels were observed when samples were treated with anti-PD-1 antibodies over a period of 72 hours (**Figures 1A, B**). We observed that PD-1 expression was gradually reduced from steady state when treated with all three antibody conditions, the most rapid effect seen with the combination therapy. Combination therapy with pembrolizumab and NB01 significantly reduced surface PD-1 levels at 6 hours of incubation and reached peak surface PD-1 downregulation by 24 hours for memory CD4 and CD8. All three antibody conditions reached significant downregulation by the 24-hour time point in memory CD8 in comparison to the IgG4 control.

PD-1 surface expression steadily dropped for the following time points before plateauing to approximately 18% surface expression on both memory CD4^+^ and CD8^+^ for the combination therapy, representing over a 71% decrease from baseline (**Figure 1C**). Individually, pembrolizumab reached to 61% decrease in PD-1 expression and NB01 was seen with a 50% decrease compared to the IgG4 control by 72 hours. While anti-PD-1 antibodies were effective in reducing the overall expression of PD-1, a complete abrogation of PD-1 expression did not occur. Furthermore, total memory CD4^+^ and CD8^+^ T cells frequencies as well as the proportion of live CD3^+^ cells remained steady across antibody conditions, confirming that the depletion of PD-1 expressing T cells was not a factor for PD-1 receptor downregulation (**Supplementary Figure 1**). The addition of anti-PD-L1 in parallel with pembrolizumab or IgG4 control had no impact on the downregulation of PD-1 expression, which indicated that the blockade of PD-1 and PD-L1 interaction was not a contributing factor to the waning of PD-1 surface expression on memory T cells (**Supplementary Figure 2**).

### Surface PD-1 downregulation kinetics occurs at a slower rate than anti-PD-1 antibody internalization and requires the presence of effector cells within blood mononuclear cells

**Figure 2:**
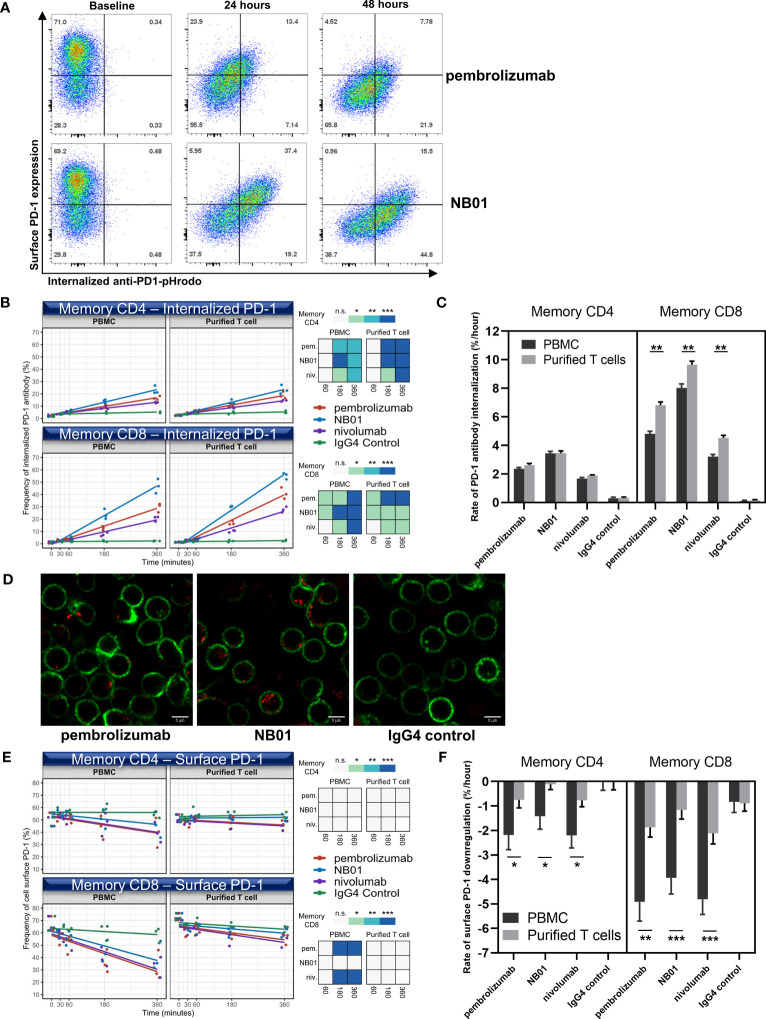
Internalization of PD-1 antibodies and the downregulation of PD-1 surface receptor induced by anti-PD-1 antibodies occur at different rates and surface PD-1 downregulation is contingent on the presence of effector cells. **(A)** PD-1 surface expression vs internalized anti-PD-1 antibody measured over 24 and 48 hours in memory CD8. Early time-points (0, 30, 60, 180, and 360 minutes) measured for internalized PD-1 antibody **(B)** and surface PD-1 downregulation **(E)** in memory T cells within total blood mononuclear cells (PBMC) or purified T cell composition using pembrolizumab, nivolumab, NB01, or IgG4. (n = 3 donors, shown as averaged triplicates with line of best-fit) Heatmap representation of significance: * denotes p ≤ 0.05; ** denotes p ≤ 0.01; *** denotes p ≤ 0.001 in two-tailed unpaired *t* test with Welch’s correction compared to IgG control. **(D)** Live cell imaging of blood mononuclear cells incubated for 6 hours with pHrodo conjugated pembrolizumab, NB01, or IgG4 (red) stained with CD3 (green) for total T cell detection and representative of 4 independent experiments **(C, F)** Kinetic rate of antibody internalization and rate of surface receptor downregulation. Kinetics data measured by linear regression analysis and represented as mean ± SE on combined replicates from 3 donors.* denotes p ≤ 0.0421; ** denotes p ≤ 0.01; *** denotes p ≤ 0.0008 in two-tailed unpaired *t* test with Welch’s correction. n.s. denotes non-significant.

In order to determine if antibody induced surface PD-1 downregulation was related to the internalization of antibody-receptor complex, we used pHrodo fluorophores conjugated to antibodies to quantify the total amount of endocytosed anti-PD-1 antibodies. The pHrodo compound acts as a pH indicator, remaining undetectable with low background at neutral pH and increasing in fluorescence as acidity in its immediate environment increases. The validation of pHrodo sensitivity was assessed using OKT3-pHrodo for CD3 internalization (**Supplementary Figure 3**). pHrodo conjugation was performed on pembrolizumab, NB01, nivolumab, and IgG4 isotype control antibodies and the conjugated antibodies performed similarly to their respective unconjugated antibody formats in terms of surface PD-1 downregulation (data not shown). We were able to simultaneously measure surface PD-1 downregulation and anti-PD-1 antibody internalization by flow cytometry at 24- and 48-hour time points for pHrodo-conjugated pembrolizumab and NB01 (**Figure 2A**).

We chose to observe earlier timepoints given that endocytotic events are typically rapidly induced and occur within minutes to hours. We also compared two cell type compositions, total blood mononuclear cells and purified T cells, to see if either downregulation and/or internalization was T cell intrinsic or required the help of bystander effector cells. Kinetic rates of anti-PD-1 antibody internalization and surface PD-1 downregulation were measured by linear regression analysis and represented as a line of best fit.

In the first hour, the anti-PD-1 antibodies did not show major increases in the total amount of internalized antibody in memory CD4 but antibody internalization occurred for pembrolizumab and NB01 in memory CD8 compared to the IgG4 control (**Figure 2B**). From 3-6 hours, all three anti-PD-1 antibodies were significantly internalized in both memory CD4^+^ and CD8^+^ T cells and a clear divergence was shown between the anti-PD-1 antibodies with NB01 having the highest total amount internalized followed by pembrolizumab and nivolumab. The rate of internalization per hour for each antibody condition in total blood mononuclear cells was measured: NB01 3.4%/hr and 8.0%/hr, pembrolizumab 2.3%/hr and 4.8%/hr, and nivolumab 1.6%/hr and 4.5%/hr for memory CD4^+^ and CD8^+^ T cells, respectively (**Figure 2C**). Purified T cells also showed a significant increase in the rate of internalization for memory CD8^+^ T cells but not in memory CD4, indicating that the memory CD8 were able to internalize anti-PD-1 antibodies at a faster rate in the absence of other total blood mononuclear cells.

We visually inspected the quantity of internalized antibody-vesicles in live CD3^+^ T cells determined by confocal microscopy after 6 hours (**Figure 2D**). The increased antibody internalization for NB01 antibody that was previously observed by flow cytometry was shown as more vesicles per PD-1^+^ CD3^+^ T cells compared to pembrolizumab.

In contrast to the internalization data, surface PD-1 downregulation showed that pembrolizumab and nivolumab had a greater effect in reducing surface PD-1 levels while NB01 had a lesser effect. The rate of surface PD-1 downregulation per hour for each antibody condition in total blood mononuclear cells: NB01-1.4%/hr and -3.9%/hr, pembrolizumab -2.1%/hr and -4.9%/hr, nivolumab-2.2%/hr and -4.8%/hr for memory CD4^+^ and CD8^+^ T cells, respectively (**Figure 2E**). Furthermore, the memory CD8 within the total blood mononuclear cells condition was able to significantly downregulate surface PD-1 for pembrolizumab and nivolumab at 3-6 hours but purified T cells were severely obstructed in downregulating PD-1 for all three anti-PD-1 antibodies for both memory CD4 and CD8, indicating that an effector population was necessary for the downregulation effect that was removed upon the isolation of CD3^+^ T cells (**Figure 2F**).

### Inhibition of dynamin and actin mobilization prevents both surface PD-1 downregulation and anti-PD-1 antibody internalization in memory CD8^+^ T cells

**Figure 3:**
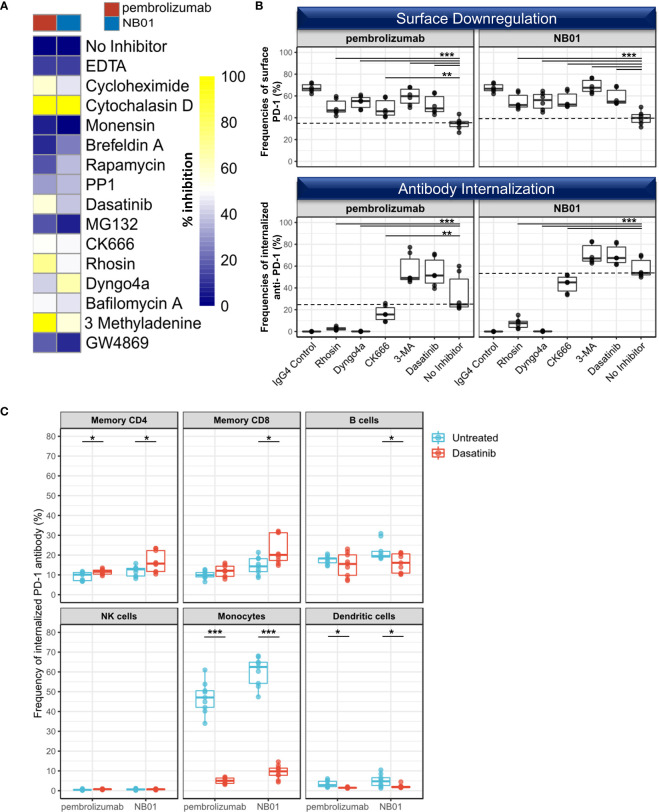
Selective inhibiton of dynamin/actin-mediated processes disrupts PD-1 antibody internalization on CD8 T cells **(A)** Heatmap of average surface PD-1 expression after 24 hours of anti-PD-1 incubation with a panel of 15 inhibitors targeting protein internalization, transport, and degradation pathways in memory CD8^+^ within total blood mononuclear cells and representative of 3 independent experiments **(B)** Selected inhibitors used to simultaneously measure the inhibition of surface PD-1 downregulation and the inhibition of PD-1 antibody internalization in memory CD8. (n = 3 donors, duplicates, dotted line representing median levels of no inhibitor control) Individual data points shown as a box plot with whiskers from minimum to maximum value. ** denotes p ≤ 0.0044; *** denotes p ≤ 0.0009 in two-tailed unpaired *t* test with Welch’s correction compared to no inhibitor control. **(C)** Pulse chase experiment with anti-PD-1-pHrodo bound to T cells in the presence of FcR block, removed from supernatant, reconstituted with unlabeled PD-1 antibodies, and incubated for 24 hours in the presence or absence of dasatinib. Uptake and internalization of anti-PD-1-pHrodo measured in different immune subsets (memory CD4, memory CD8, B cells (CD19^+^), NK cells (CD56^+^), monocytes (CD14^+^), and dendritic cells (Lin^-^ CD14^-^ HLA-DR^+^) (n=3, triplicates). Individual data points shown as a box plot with whiskers from minimum to maximum value. * denotes p ≤ 0.05; *** denotes p ≤ 0.001 in two-tailed unpaired *t* test with Welch’s correction.

We sought to determine the pathways involved in surface PD-1 downregulation and used a diverse panel of chemical inhibitors targeting endocytosis, actin mobilization, protein degradation, and transport pathways (**Figure 3A**). The selected inhibitors alone showed no significant modulation of homeostatic cell surface PD-1 expression on memory T cells and minimal cytotoxicity at the titrated concentrations used (**Supplementary Figure 4**). Inhibition of dynamin mediated endocytosis (dyngo4a) as well as actin mobility (cytochalasin D, CK666, rhosin) and lysosomal activity (bafilomycin A) were all shown to prevent antibody induced surface PD-1 downregulation in memory CD8^+^ T cells. Protein synthesis inhibitor cycloheximide was also shown to inhibit surface PD-1 downregulation, which can be attributed to pleiotropic effects on cytoskeletal rearrangement. Finally, we found that Src inhibitors dasatinib and PP1 as well as 3-MA, a class I/III PI3K inhibitor, were able to prevent surface PD-1 downregulation on memory CD8^+^ T cells.

In contrast, proteasomal degradation, recently described as the physiological route for PD-1 receptor turnover (18) and inhibited by MG132 as well as broad MMP inhibition with EDTA, exosome secretion inhibition with GW4869, mTOR inhibition with rapamycin, and protein transport inhibition with monensin or brefeldin A all had a non-significant or no effect on surface PD-1 downregulation.

We further investigated five of these inhibitory compounds (rhosin, dyngo4a, CK666, 3-MA, dasatinib) to verify their effects on surface PD-1 downregulation and anti-PD-1 antibody internalization simultaneously using the pHrodo conjugated anti-PD-1 antibodies. Here, we observed that rhosin, dyngo4a, and CK666 were able to effectively prevent both surface PD-1 downregulation and anti-PD-1 antibody internalization in the memory CD8^+^ populations (**Figure 3B**). On the other hand, dasatinib and 3-MA inhibited surface downregulation of PD-1 but had no inhibitory effect on the internalization of anti-PD-1 antibody and rather increased the total amount of internalized anti-PD-1 antibody in memory CD8^+^ compared to the no inhibitor control, indicating a selective inhibition of T cell extrinsic factors such as effector cells may be responsible for surface PD-1 downregulation rather than endocytosis (**Figure 3B**).

We selected dasatinib for further examination on the uptake of anti-PD-1 antibodies in different blood mononuclear cell populations (memory CD4^+^, memory CD8^+^, B cells, NK cells, monocytes, and dendritic cells) in a pulse-chase experiment using pHrodo-conjugated anti-PD-1 antibodies preloaded onto T cells in the presence of FcγR block, the supernatant washed, and replaced with their respective unlabeled antibodies.

In untreated blood mononuclear cells, the amount of internalized anti-PD-1 antibodies was found to be the highest in the monocyte population (HLA-DR^+^ CD14^+^ CD11b^+^), indicating that this was likely to be the main effector population involved for the removal of anti-PD-1 antibody bound on memory T cells (**Figure 3C**). We also found that B cells (CD19^+^) and to a small extent, dendritic cells (Lin^-^ CD14^-^ HLA-DR^+^) were also involved in the uptake of T cell bound anti-PD-1 antibodies. With the dasatinib treatment, monocyte mediated anti-PD-1 antibody transfer and internalization was drastically reduced and represented the highest proportional loss (89.5% and 84.3% reduction for pembrolizumab and NB01, respectively), signifying that the unaffected surface PD-1 expression on memory T cells under dasatinib were due to the inhibition of monocytic activity in antibody uptake. Moreover, this loss of the surface PD-1 downregulation effect induced by dasatinib treatment resulted in significantly increased anti-PD-1 internalization for both anti-PD-1 antibodies in memory CD4 and NB01 for memory CD8. The cross-linking of antibody-immune complexes and the FcγR produces endocytic/phagocytic responses that requires the recruitment of Src kinases and activation of Syk in myeloid and granulocytic cells (35–37). Dasatinib, which was initially designed as a kinase inhibitor against Bcr-Abl for myeloid leukemia and gastrointestinal stromal tumors, affects a wide range of targets within the Src family kinases upstream of SFK and is known to inhibit FcγR function (38–40).

### Antibody induced surface PD-1 downregulation requires the presence of monocytes and is mediated by the binding of anti-PD-1 antibody Fc domain with Fc receptor CD64

**Figure 4:**
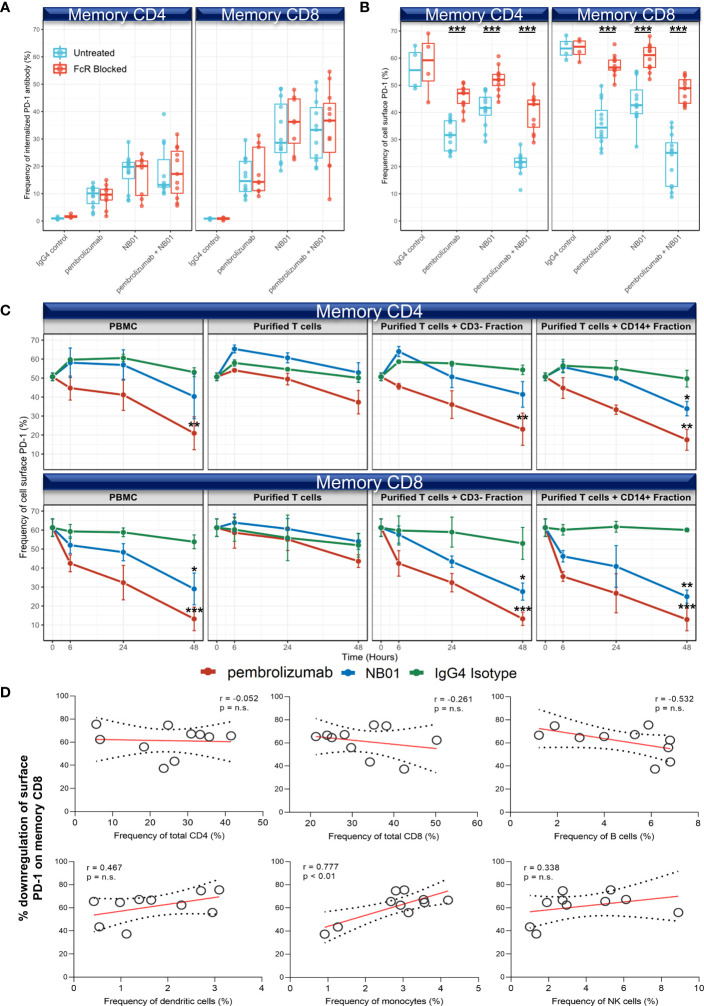
Surface PD-1 downregulation by anti-PD-1 antibodies is mediated primarily through the crosslinking of bound PD-1 antibody Fc region with FcγR on monocytes. **(A, B)** Fc receptor blockade with 50 μg/mL of human IgG and anti-CD32/CD64 was measured for internalized PD-1 antibody **(A)** and surface PD-1 expression **(B)** after 24 hours of incubation (n = 4 donors, triplicates). Individual data points shown as a box plot with whiskers from minimum to maximum value. *** denotes p ≤ 0.00008 in unpaired *t* test with Welch’s correction. **(C)** Antibody mediated downregulation kinetics measured in blood mononuclear cells (PBMC), purified T cells, and purified T cells reconstituted with autologous CD3- or CD14+ fraction (5:1 ratio) (n = 3 donors, one-way ANOVA). Data represented as mean ± SD. * denotes p ≤ 0.05; ** denotes p ≤ 0.01; *** denotes p ≤ 0.001 in two-tailed unpaired *t* test with Welch’s correction compared to IgG control at 48 hours. **(D)** Pearson’s correlation on chronic HIV infected donors for the % decrease of surface PD-1 induced by combined pembrolizumab and NB01 antibodies relative to blood mononuclear subsets after 24 hours (n = 10 donors). n.s. denotes non-significant.

Given that the blood mononuclear cell populations (monocytes, B cells, and dendritic cells) that uptake anti-PD-1 antibodies have high expression of Fcγ receptors, we next asked whether the interaction of surface PD-1 downregulation was contingent on availability of Fc receptors for binding the Fc domain of the anti-PD-1 antibodies. We found that FcγR blockade in the presence of anti-PD-1 antibodies had no impact on the internalization of anti-PD-1 antibodies in memory T cells (**Figure 4A**). On the other hand, FcγR blockade had a drastic effect on the downregulation of surface PD-1 for all three antibody conditions, demonstrating that the activity of FcγR interaction is necessary to observe surface PD-1 downregulation on memory T cells. (**Figure 4B**)

Next, we performed the surface PD-1 downregulation experiment over 48 hours using total blood mononuclear cells, purified T cells, and purified T cells reconstituted with their autologous CD3^-^ depleted or CD14^+^ fractions at a 5:1 ratio. Purified T cells did not have a significant reduction in surface PD-1 expression compared to its IgG4 control for both memory CD4 and CD8 T cells as previously observed. Reconstitution of autologous CD3^-^ and CD14^+^ populations significantly restored surface PD-1 downregulation in both memory CD4 and CD8 T cells by 48 hours to levels similar to the total blood mononuclear cells control, confirming that the effector cell populations in the CD3^-^ depleted or CD14^+^ fractions were necessary for PD-1 surface downregulation (**Figure 4C**).

We further checked in ten HIV^+^ donors for the percent downregulation of surface PD-1 under anti-PD-1 treatment compared against the relative frequencies of different blood mononuclear cell populations. While no significant correlations were observed in dendritic cells (r = 0.467, p =n.s.) and B cells (r = -0.532, p =n.s.), only the frequencies of monocytes were significantly correlated with surface PD-1 downregulation (r = 0.777, p < 0.01) after 24 hours (**Figure 4D**).

**Figure 5:**
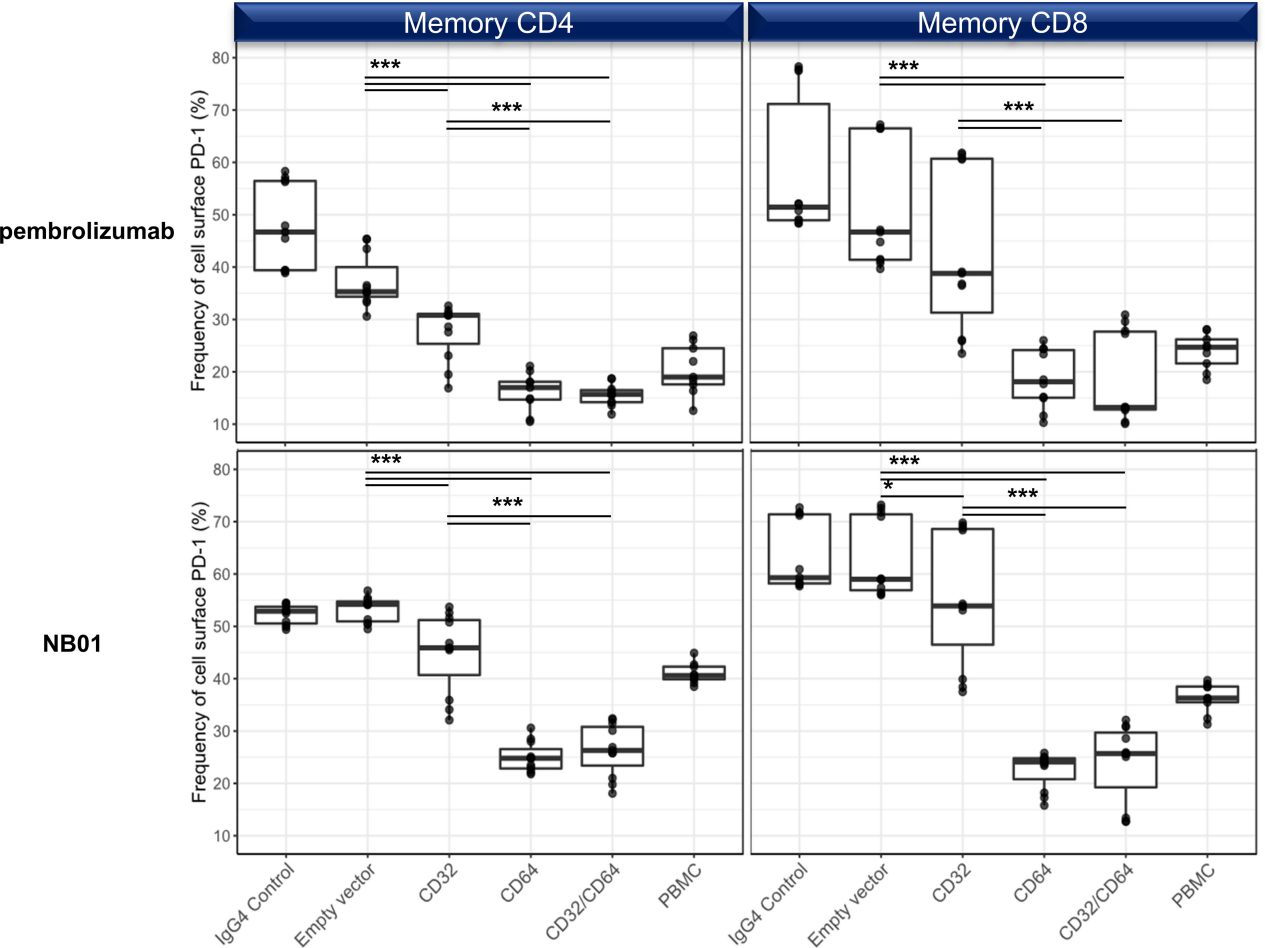
Surface PD-1 downregulation by anti-PD-1 antibodies is mediated primarily through the crosslinking with CD64 and to a lesser degree, CD32a. 293T transfection of empty vector, CD32a, CD64, or CD32a + CD64 and cocultured with purified T cells in the presence of pembrolizumab, NB01, or IgG4 control. (n = 3 donors, triplicates) Individual data points shown as a box plot with whiskers from minimum to maximum value. * denotes p ≤ 0.02161; *** denotes p ≤ 0.00006 in two-tailed unpaired *t* test with Welch’s correction.

We used transfected 293T cells expressing CD32, CD64, CD32 + CD64, or empty vector control and co-cultured with primary T cells to distinguish the Fc receptor responsible for surface PD-1 downregulation. We observed that both CD32 and CD64 strongly reduced PD-1 levels on memory T cells; however, CD64 alone had a more pronounced effect on surface PD-1 levels which was significantly more decreased than CD32 alone (**Figure 5**). The co-transfection of CD32 and CD64 did not augment the downregulation effect in comparison to CD64 alone, indicating that CD64 alone represented the bulk of this interaction. We also checked for CD16a activity using a stable transfected Jurkat cell line but this Fc receptor did not have any impact on surface PD-1 downregulation (**Supplementary Figure 5**). Lastly, we verified whether ADCP was a factor in the downregulation of PD-1 in memory T cells by using autologous monocytes and M1-/M2- differentiated macrophages. None of the anti-PD-1 antibodies increased the proportion of phagocytosed T cells relative to the IgG4 control (**Supplementary Figure 6**).

### FcR-mediated anti-PD-1 surface downregulation enhances CD8^+^ T cells proliferation under antigen-specific stimulation

**Figure 6:**
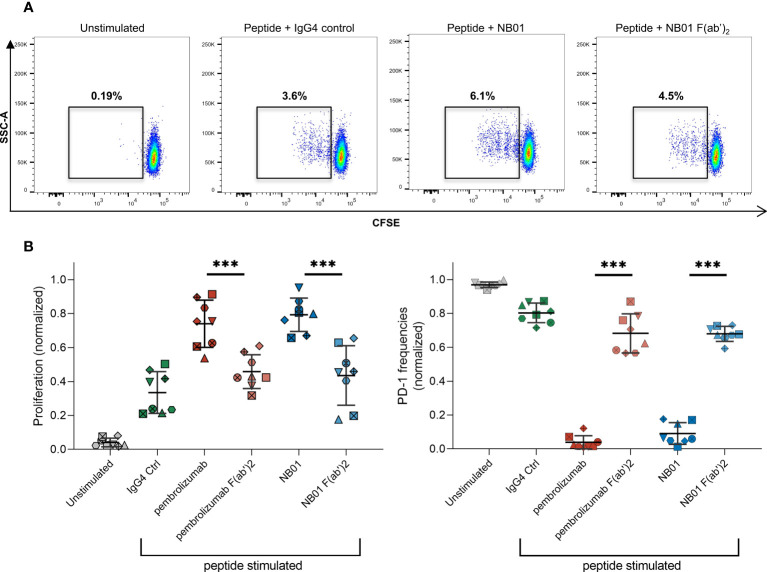
Standard anti-PD-1 antibodies boost antigen-specific T cell proliferation compared to their F(ab’)_2_ variants. **(A)** Frequencies of proliferating CFSE^lo^ memory CD8 T cells in different antibody conditions stimulated with pooled Gag PTE peptides for 5 days. **(B)** Proliferation capacity and PD-1 expression in memory CD8 with different antibody conditions. (n = 8 donors, averaged of 3-4 replicates with data normalized to each donor) Data represented as mean ± SD. *** denotes p < 0.0005 in two-tailed unpaired *t* test with Welch’s correction.

We next addressed the functionality of PD-1 downregulation under antigen-specific conditions. F(ab’)_2_ variants of pembrolizumab and NB01 were generated to make antibodies that retained antigen binding sites but lacked Fc effector binding. In a five-day stimulation experiment using pooled HIV-1 Gag peptides, significantly higher proliferation was observed for all standard antibody conditions in memory CD8^+^ T cells compared to their respective F(ab’)_2_ variants (**Figures 6A, B**). Full format pembrolizumab and NB01 were significantly increased in proliferation compared to the peptide + IgG4 control; pembrolizumab F(ab’)2 was also able to reach significance (p = 0.0458) but NB01 F(ab’)2 was not significantly increased (p = 0.2099) compared to peptide + IgG4 control. Furthermore, PD-1 expression was significantly decreased when using the standard format of the anti-PD-1 antibodies compared to their respective F(ab’)_2_ variants. Of note, the PD-1 F(ab’)_2_ antibodies were also able to significantly reduce PD-1 expression (pembrolizumab F(ab’)_2_: p = 0.0238/NB01 F(ab’)_2_: p = 0.0003) compared to the IgG4 control but to a much lesser degree than the full format antibodies. The lower total availability of PD-1 receptor during antigenic stimulation is likely to drive the increased proliferative capacities of exhausted PD-1^+^ CD8^+^ T cells. Given that the anti-PD-1 F(ab’)_2_ antibodies were unable to efficiently reduce PD-1 expression, their impact on antigen-specific CD8^+^ T cells proliferation was shown to be reduced compared to their respective full antibodies.

## Discussion

In this study, we have shown that anti-PD-1 antibody internalization and PD-1 surface downregulation occur as distinct processes. To our knowledge, this is the first reported study showing primary human T cells engaging in the active internalization of anti-PD-1 antibodies. We also shown that the standard IgG4 format of therapeutic anti-PD-1 antibodies is necessary to engage Fc receptors on monocytes and that the downregulation of PD-1 has a distinct effect in restoring T cell functionality. Both blocking and non-blocking anti-PD-1 antibodies performed similarly, indicating that the physical removal of PD-1 from the cell surface plays a major role in enhancing T cell activity rather than blockade alone.

The internalization of anti-PD-1 antibodies in memory T cells was shown to be more rapidly induced and occurred regardless of the composition of its surrounding cells in a T cell intrinsic process. While antibody internalization could possibly be attributed to intrinsic surface PD-1 turnover, we did not observe any increases in surface PD-1 expression with MG132 inhibition of the proteasome compared to the no inhibitor control, indicating that proteasomal receptor degradation was not a factor in regulating PD-1 surface expression. This was in contrast with a previous report that showed PD-1 surface expression increases when the proteasomal degradation pathway was blocked (18). However, the regulation of activation induced PD-1 expression under stimulation conditions compared to the physiological PD-1 exhaustion profile acquired in *in vivo* in the course of chronic viral infection that we used in this study may explain this disparity.

The downregulation of total surface PD-1 expression in memory T cells occurred more slowly, reaching its peak at 24-48 hours and required the presence of Fc receptor expressing cells. Monocytes were shown to be the cell subset with the highest impact for the surface PD-1 downregulation effect on T cells and increased monocyte frequencies positively correlated with the degree of surface PD-1 downregulation. This downregulation effect was observed with both pembrolizumab and nivolumab classical blocking anti-PD-1 mAbs currently marketed for cancer immunotherapy as well as a novel anti-PD-1 antibody the we previously reported that relieves functional T cell exhaustion through a mechanism that does not require the blockade of PD-1 with its cognate ligands (15, 41). Numerous studies have shown that with monoclonal antibody therapies targeted against CD4, CD20, CD25, CD38, HER2, CTLA-4, PD-1, and PD-L1, FcγR-expressing effector cells such as monocytes, macrophages, and granulocytes imparted a significant effect onto target cells by the crosslinking of the Fc domain of target antibody bound to its antigen and an FcγR *in trans* (42–50). In particular, the effects of anti-CD20 antibodies on downregulating surface CD20 expression has been heavily investigated over the past decade. A study by Dahal, L. et al. showed that the internalization and the downregulation of anti-CD20 antibodies occurred as separate processes whereby the downregulation of anti-CD20 on opsonized B cells occurred as a byproduct of phagocytotic events by macrophages in conditions where phagocytic capacity was inhibited due to bead uptake and saturation (51). Moreover, the authors found that obinutuzmab, a type II anti-CD20 antibody with low internalization capacity, had increased receptor downregulation in primary CLL samples mediated by monocyte-derived macrophages. The culmination of these reports show that antibody mediated receptor downregulation represents an underappreciated but consistently observed effect that has the potential to dictate the outcomes in mAb therapies.

In our work, we have clearly shown that the availability of FcγR binding was critical in observing surface PD-1 downregulation as shown by the FcγR blockade experiments. Additionally, the transfection studies revealed that the FcγRI/CD64 was primarily responsible for producing this effect. CD64 is mainly expressed on monocytes/macrophages and monocyte-derived DCs while inducible in neutrophils, eosinophils, and mast cells and therefore, the likely mediators of this interaction by homeostatic abundance would be the monocytic population (52). In the work by Kreig, C, et al., higher frequencies of CD14^+^ CD16^-^ HLA-DR^+^ CD64^+^ monocytes were shown to be a positive indicator for anti-tumor responses in anti-PD-1 therapy measured by progression-free survival and overall survival in responders compared to non-responders in stage IV melanoma (53). Another study by Olingy, C showed that CD33^hi^ monocytes predicated responsiveness to PD-1 therapy in NSCLC patients (54). While these authors did not conclude on the mechanism that drove this finding, we believe that antibody induced surface PD-1 downregulation linked with higher monocytic frequencies may play a role in this observation.

We also showed that CD32a was capable of inducing surface downregulation but the effects were significantly lower in comparison to CD64. CD32a is typically coexpressed with CD64 on monocytes/macrophages and expressed in neutrophils and dendritic cells. CD32a has been previously implicated in other studies for mediating the downregulation of anti-CD20, anti-HER2, and anti-EGFR bound surface receptors by neutrophils, which constitutively express high levels of CD32a and CD16b (55, 56). Furthermore, CD64 expression is upregulated on activated neutrophils driven by inflammatory cytokines such as IFNγ and G-CSF or in the presence of bacterial infections (57). Neutrophils are shown to play major roles in FcR mediated function for mAb therapies (58, 59) and the relative abundance of neutrophils is likely to contribute to the downregulation of surface receptors bound by antibodies.

One of the limitations of this study was the potential off-target effects of the inhibitors used. To help mitigate this risk, a broad panel of inhibitors was used to encompass the different phases of endocytosis, protein production, and protein secretion. This inhibition of endocytic pathways with high redundancy and overlap was designed to control for off-target effects; however, we acknowledge that it cannot account for all possible interactions. Specifically, the tyrosine kinase inhibitor dasatinib is known to have a wide range of targets beyond Bcr-abl including Src family kinases such as Fyn, FAK, and Lck as well as PDGF-R and c-KIT (60). Dasatinib has been shown to act as an inhibitor for Lck in T cell activation/proliferation and is currently under investigation for the reversible inhibition of CAR-T cell tonic signaling (38, 61, 62). Interestingly, dasatinib has also been shown to target the tyrosine kinases (Fyn, Lyn, Syk) involved in FcγR function and signaling (40, 63–65). We showed that dasatinib treatment had no effect on T cell internalization of anti-PD-1 antibodies but dramatically reduced anti-PD-1 uptake and internalization in monocytes. Surface PD-1 downregulation on T cells was inhibited by dasatinib; however, given that the endocytosis of anti-PD-1 bound on T cells remained unaffected despite Lck inhibition, the loss of surface PD-1 downregulation effect can be linked to the inhibition of monocyte FcγR function.

There are reports that show that anti-PD-1 antibodies are affected by Fc-FcγR interactions and other groups have evaluated detrimental effects in the functional activity of anti-PD-1 antibodies when engaging Fc receptors on macrophages specifically in the tumor microenvironment. Dahan, R et al. found that the knockout of CD64 expression enhanced anti-tumor responses when using chimeric anti-PD-1 antibodies [23]. In another study, Arlauckas and colleagues showed reduced treatment efficacy with anti-PD-1 transfer and uptake by macrophages in MC38 adenocarcinoma mouse model (66). While the specific Fc receptors involved were not clearly explicated, experiments with Fc receptor blockade or F(ab’)_2_ antibodies showed tumor size reduction in an *in vivo* model. This stood in contrast with our results which showed a significantly increased functional recovery of memory CD8^+^ T cells when using the complete mAb format over its respective F(ab’)_2_ variant. This discrepancy may be related to an issue of commonly used anti-mouse PD-1 antibodies leading to a depletion of antigen-specific T cells in mouse models and differing in functional activity compared to clinical modalities (67, 68). Moreover, a recent report by Ye, D. et al. found that a high density of proinflammatory CD64^+^ macrophages in close proximity with CD8 in the tumor microenvironment correlated with increased median overall survival in patients with multiple cancer types undergoing PD-1 therapy (69).

The main limitation of our study was the use of an *ex vivo* system with monocytes as the primary mediator of this interaction and thereby losing the effect of the cellular and cytokine composition of the tissue environment. There has been an insufficiency of conclusive *in vivo* studies showing the antibody-mediated downregulation effect. An early study by Li, Yong et al. showed that injection of rituximab in SCID mice with Z138 tumors resulted in the significant loss of CD20 expression that was inhibited by the administration of IVIG (70). Other clinical studies observed the surface downregulation of target receptors such as CD20, CD25, and CD38 in patients treated with mAb therapy and correlated to *ex vivo* experiments (44, 47, 71). However, there is still a pending need to validate the effects of antibody-mediated downregulation through *in vivo* systems in order to accurately assess the impact and implications of this mechanism in mAb therapy.

The use of human anti-PD-1 antibodies with bona fide, functionally exhausted PD-1 high human primary cells in our study represents a simpler yet relevant model to reveal the possible human Fc-FcR interactions in PD-1 therapy that can be further investigated. We have shown that with antigen-specific stimulation of exhausted memory CD8^+^ T cells, FcγR engagement and subsequent downregulation of PD-1 is superior for recovering proliferation than PD-1 blockade alone. It should be noted that the functional enhancement of exhausted T cells through immune checkpoint inhibitors such as anti-PD-1 does not necessarily indicate that the exhaustion phenotype has been reversed given the stable transcriptional profile and epigenetic remodeling of the terminally exhausted T cell subset; the mechanisms regulating the complete reversal of T cell exhaustion are currently the focus of extensive investigation (72–75).

The results presented in this report identify a novel and alternative, FcR mediated mechanism by which anti-PD-1 antibodies can downregulate the expression of surface PD-1 on memory T cells and contribute to the PD-1/PD-L1 blockade to promote optimal recovery of effector functions in exhausted T cells. While all anti-PD-1 antibodies tested were able to effectively internalize in memory T cells, the downregulation of the PD-1 receptor itself was reliant on CD64 expressing monocytes, leading to implications for the prediction of efficacy in anti-PD-1 treatment. The present observations support a model in which the FcR mediated downregulation of the surface expression of PD-1 on effector CD8+ T cells hijacks PD-1 from the inhibitory signal delivered by its ligand PD-L1, potentially resulting in a more potent and durable anti-PD-1 therapeutic efficacy. Thus, our findings emphasize the necessity of understanding the interactions of PD-1 mAb therapy and further investigations are needed to distinguish the influence of CD64 and other potential FcRs mediated effects for PD-1 therapy in clinical settings.

## Materials and methods

### Study donors and cell lines

Sixteen HIV+ viremic donors were used throughout in this study. Blood mononuclear cells were maintained in complete RPMI (Invitrogen) supplemented with 10% heat-inactivated fetal bovine serum (FBS, Biowest), 100 U/ml penicillin (BioConcept), and 100 µg/ml streptomycin (BioConcept). 293T cells were maintained in DMEM (Invitrogen) supplemented with 10% (v/v) FBS, 100 U/ml penicillin (BioConcept), and 100 μg/ml streptomycin (BioConcept). Cells were kept at 37°C, 5% CO^2^ for the following experiments.

### Antibodies and reagents

The following antibodies were used in this study: anti-CD3 (OKT3), anti-CD4 (RPA-T4), anti-CD8 (RPA-T8), anti-CD45RA (HI100), anti-CD14 (61D3), anti-HLA-DR (G46-6), anti-CD11b, anti-CD56 (HCD56), anti-CD19 (HIB19), anti-CD16 (3G8), anti-CD32 (FUN-2), anti-CD64 (10.1), anti-PD-L1 (29E.2A3). Anti-PD-1 antibodies pembrolizumab (Merck), nivolumab (Bristol-Myers Squibb), and NB01 were used for experimental conditions and for the primary staining of PD-1 with their respective antibody condition. Anti-human IgG Fc secondary antibodies conjugated with Alexa Fluor 594 (Biolegend) or APC (Biolegend) were used for the detection of surface PD-1 expression. Preclinical grade IgG4 isotype control (Bingo Bio) was as a negative control for all anti-PD-1 experiments.

### pH-sensitive dye conjugation

200 μg of anti-PD-1 antibody or IgG4 isotype control was concentrated with Amicon^®^ Ultra-0.5 50 kDa spin filter (Millipore) at 12’000 g for 10 minutes. 100 μL of 4 mM TCEP solution (Thermo) was added to the spin column, mixed by pipetting, and incubated for 30 min at 37°C before washing two times in PBS and resuspension with PBS at 100uL. 10 mM pHrodo™ Red or pHrodo™ Green maleimide (Thermo) solution was freshly prepared with DMSO and a 10-molar ratio excess of pHrodo maleimide was added to the antibody and incubated for 2 hours at 37°C. After conjugation, antibody-pHrodo mixture was washed four times in PBS and resuspended in 200 μL of PBS followed by filter sterilization with Ultrafree-MC Centrifugal Filter 0.22 μM (Merck). Protein concentration and degree of labeling were measured by Nanodrop (Thermo).

### Surface PD-1 downregulation, anti-PD-1 antibody internalization, and proliferation assay

Blood mononuclear cells from selected donors were thawed and rested overnight at 37°C. Blood mononuclear cells were then treated with 5 μg/mL with different anti-PD-1 antibodies with or without pHrodo conjugation and 5 μg/mL of IgG4 isotype control in complete RPMI for hours to days depending on the length of the experiment. Cells were washed at each time point and stained again with the combination of blocking and non-blocking anti-PD-1 antibodies to quantify total surface PD-1 before proceeding to extracellular staining for memory T cells. Internalized anti-PD-1 antibody was measured as fluorescence intensity from pHrodo Red or Green. All flow cytometry measurements were obtained on BD Fortessa. T cell isolations was performed with EasySep™ Human T Cell Isolation Kit, CD3- fraction was isolated from the supernatant after magnetic attachment using the EasySep™ Human CD3 Positive Selection Kit II, and the CD14+ fraction was isolated using EasySep™ Human Monocyte Enrichment Kit without CD16 Depletion kit. The proliferation assays were performed as previously described (15). Briefly, blood mononuclear cells from selected donors were thawed and rested overnight at 37°C. Blood mononuclear cells were labeled with 2μM of CFSE (Sigma) and then treated with 5 μg/mL of different anti-PD-1 antibodies or F(ab’)_2_ conditions in the presence of 1 μg/mL of Gag PTE pooled peptides (NIH AIDS Reagent) for 5 days. On day 5, cells were washed and stained for flow cytometry analysis.

### Inhibitors

Chemical compounds selected for inhibiting protein endocytosis, transport, and degradation pathways were used at the following concentrations: EDTA (100 μM), cycloheximide (5 μg/mL), cytochalasin D (100 nM), monensin (2 μM), brefeldin A (10 μg/mL), rapamycin (1 μM), PP1 (20 μM), dasatinib (1 μM), MG132 (250 nM), CK666 (150 μM), rhosin (200 μM), dyngo4a (60 μM), bafilomycin A (500 nM), 3-methyladenine (5 mM), GW4869 (5 μM). Inhibitors were titrated for the highest usable concentration with minimal cytotoxicity in complete RPMI within a 24-hour incubation period for experiments. Blood mononuclear cells were pretreated with each inhibitor for 1 hour at 37°C before the addition of anti-PD-1 antibodies at 5 μg/mL and the continuation of internalization/downregulation experiments for 24 hours with subsequent surface staining followed by flow cytometry.

### Pulse-chase experiment with pHrodo-conjugated anti-PD-1 antibodies

Blood mononuclear cells were incubated with 5 μg/mL anti-PD-1-pHrodo antibodies for 30 minutes at 4°C in the presence of Human Trustain FcX block (Biolegend). Cells were washed twice and resuspended in complete RPMI containing 5 μg/mL of their respective unlabeled antibodies and incubated for 24 hours with or without dasatinib treatment at 1 μM. Surface panel for different blood mononuclear cell subset compositions were used and internalized pHrodo-antibody was detected within the subsets by flow cytometry.

### Confocal microscopy

Blood mononuclear cells were incubated with pHrodo-PD-1 or pHrodo-IgG4 antibodies for six hours at 37°C. Blood mononuclear cells were then stained with anti-CD3-APC for 20 minutes in PBS/2% FBS solution. Cells were washed, resuspended in Live Cell Imaging Solution (Thermo) and transferred to 8-well glass bottom Lab-Tek™ II Chamber Slides (Nunc) coated with poly-L-lysine, then adhered for 30 minutes at 37°C before taken for microscopy imaging. Confocal images were taken on a Zeiss LSM 880 Airyscan and image analysis was done with ImageJ.

### Fc receptor blockade

Blood mononuclear cells were set up for surface PD-1 downregulation and internalization assay as before but in the presence of 50μg/mL of human IgG4 (Sigma) and 50μg/mL of anti-CD32/CD64 (Biolegend) for unspecific and specific blocking of the Fc receptors. Blood mononuclear cells were incubated with FcR blocking antibodies for 1 hour at 37°C before the addition of pHrodo-anti-PD-1 antibodies at 5 μg/mL and the continuation of the internalization/downregulation experiments for 24 hours and subsequent surface staining followed by flow cytometry.

### 293T transfection of CD32 and CD64

Low passage HEK293T cells were seeded into 6-well plates and kept overnight at 37°C for attachment. Plasmids for CD32a and CD64 (Sino Biological) were incorporated into Fugene HD transfection reagent (Promega), resuspended in OptiMEM (Invitrogen) and added onto confluent cells for 6 hours. Cells were washed and resupplied with complete DMEM and kept in the incubator for 24 hours before detachment and seeding into flat-bottom 96 wells. Purified primary T cells were isolated as previously described above and added at a 1:2 ratio (293T: T cells) and cocultured for 48 hours before extracellular staining and FACS analysis.

### F(ab’)_2_ preparation

Anti-PD-1 antibodies were cleaved using a modified IdeS protease FabRICATOR (Genovis) into F(ab’)_2_ and Fc fragments at 1 unit per μg of antibody for 30 minutes at 37°C and buffer exchange was done using 50 kDa Amicon^®^ spin filters (Merck). Free Fc fragments were removed using Pierce™ protein A columns (Thermo) and purity was assessed by SDS-PAGE/Coomassie blue.

### Macrophage polarization and ADCP assay

Primary monocytes from healthy donors were enriched and plated into 6-well plates at 1 million cells per mL. For M1 polarization, GM-CSF was added at 50 ng/mL and for M2 polarization, M-CSF was added at 50 ng/mL for 3 days before washing and supplementing for an additional 3 days. Afterwards, M1- and M2-polarized macrophages were further activated for an additional 2 days. M1 macrophage activation was induced by GM-CSF (50 ng/mL), IFN-γ (20 ng/mL), and LPS (20 ng/mL) while M2 macrophage activation was induced by M-CSF (50 ng/mL) and IL-4 (50 ng/mL). Autologous primary T cells were isolated and stained with CFSE/CTFR before co-culture with differentiated macrophages at 5:1 ratio. Anti-PD-1 antibodies were added at 5 μg/mL and ADCP was measured in CD11b^+^ CD14^+^ macrophages 24 hours later by flow cytometry.

### Statistical analyses

Statistical significance was obtained either using a two-tailed, unpaired parametric t-test analysis with Welch’s corrections for comparison of frequencies or by one-way analysis of variance (ANOVA) for multiple group comparisons with Bonferroni’s correction. Linear regression was used to calculate slope kinetics and Pearson’s correlation test was used for correlation coefficients.

## Data availability statement

The original contributions presented in the study are included in the article/**Supplementary Material**, further inquiries can be directed to the corresponding authors.

## Ethics statement

The study was approved by the Institutional Review Board of the Centre Hospitalier Universitaire Vaudois and all individuals gave written informed consent.

## Author contributions

VJ conceived the study, performed the experiments, analyzed, and interpreted data. PC and LL developed and implemented the assay for tissue imaging and image processing. VJ, PC, AN, and MO reviewed the data analysis and provided feedback for the manuscript. CF and GP supervised and guided the study and data interpretation. VJ, CF, and GP wrote the paper and all authors reviewed and approved the manuscript. All authors contributed to the article and approved the submitted version.
