# Supplementary material for: A CD64/FcγRI-mediated mechanism hijacks PD-1 from PD-L1/2 interaction and enhances anti-PD-1 functional recovery of exhausted T cells
Source: Front Immunol. 2023 Aug 9;14:1213375. doi: 10.3389/fimmu.2023.1213375 (PMC10446174; doi:10.3389/fimmu.2023.1213375)
Supplement: Supplementary file 1 [file DataSheet_1.pdf]

Supplementary Figure 1

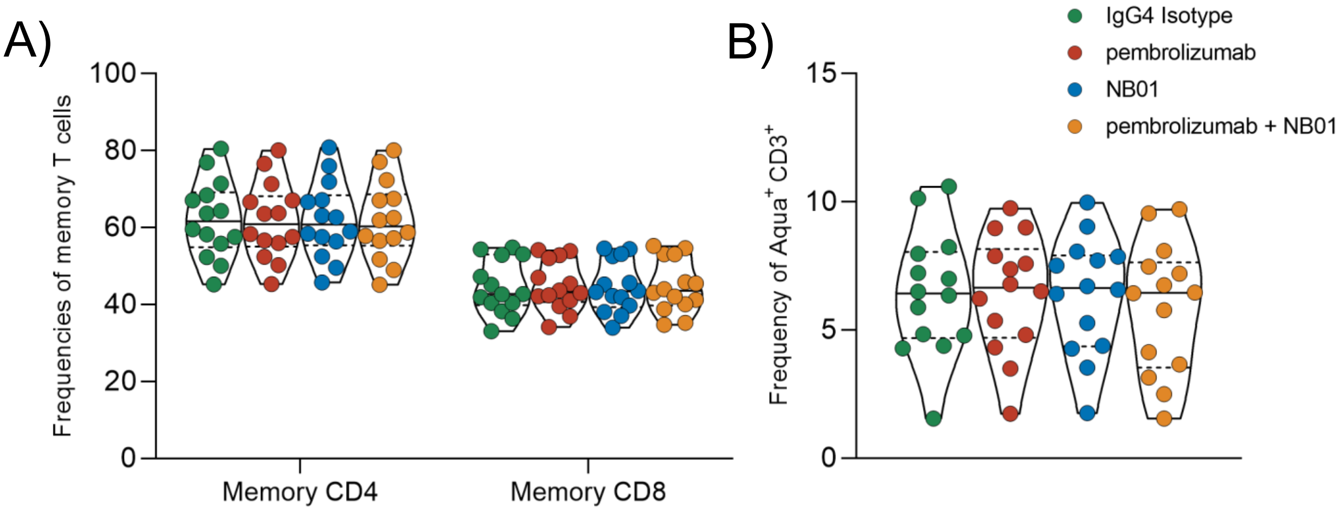

Supplementary Figure 1
Total cell events and ADCC activity after anti-PD-1 treatment for 24 hours. (A) Frequencies of viable memory CD4 and CD8 within total CD4 and CD8 respectively were measured after 24 hours with different antibody treatments. **(B)** Frequencies of dead T cells within total T cells indicating ADCC ($CD3^+$ $Aqua^+$) were measured after 24 hours with different antibody treatments. (n = 14 donors) Data represented as violin plots with median, 1st, and 3rd quantiles.

Supplementary Figure 2

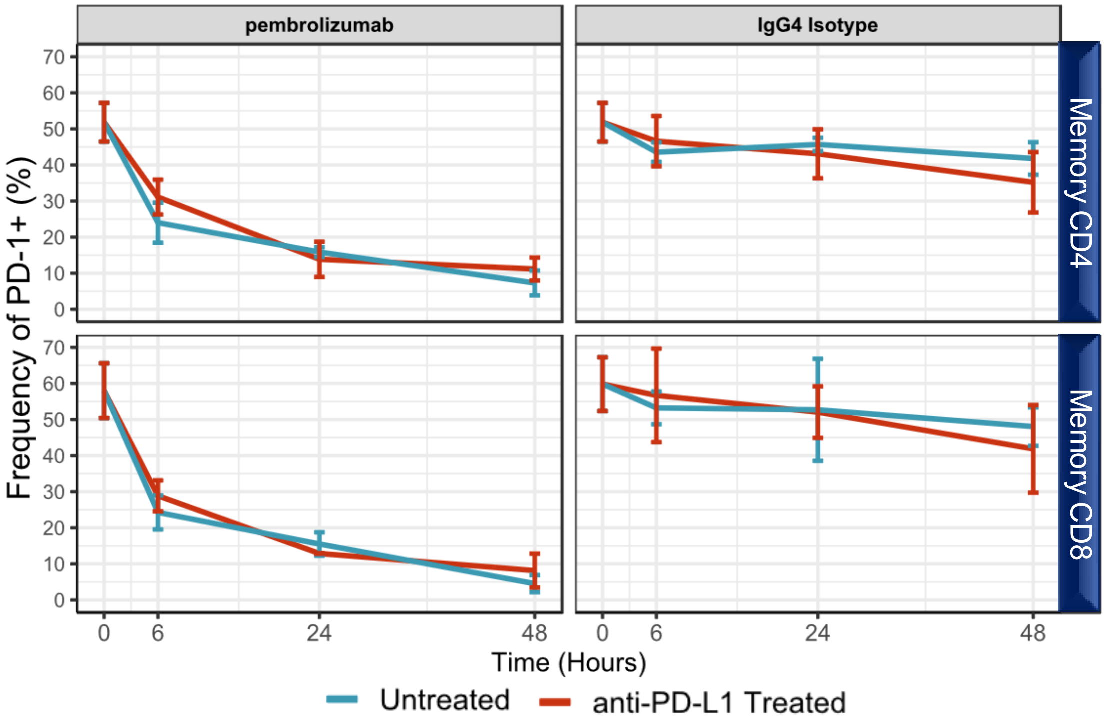

Supplementary Figure 2

Effect of anti-PDL1 combined with anti-PD-1 on PD-1 downregulation. Blood mononuclear cells were incubated with pembrolizumab or IgG4 (5 µg/mL) with or without anti-PD-L1 (5 µg/mL) for 0, 6, 24, and 48 hours. (n = 4 donors) Data represented as mean ± SE.

Supplementary Figure 3

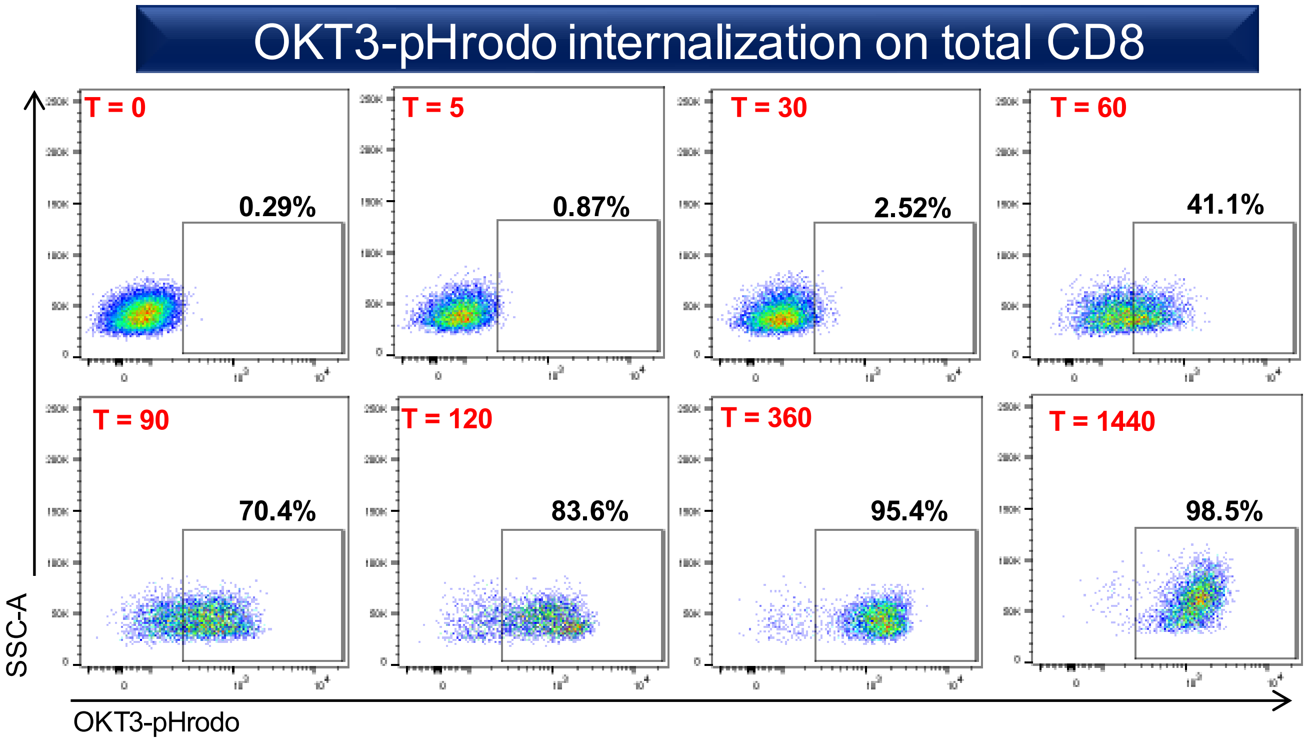

Supplementary Figure 3
Validation of pHrodo sensitivity using OKT3-pHrodo for CD3 internalization. Total $CD8^+$ T cells were incubated with anti-CD3 (5μg/mL) for 0, 5, 30, 60, 90, 120, 360, and 1440 minutes.

Supplementary Figure 4

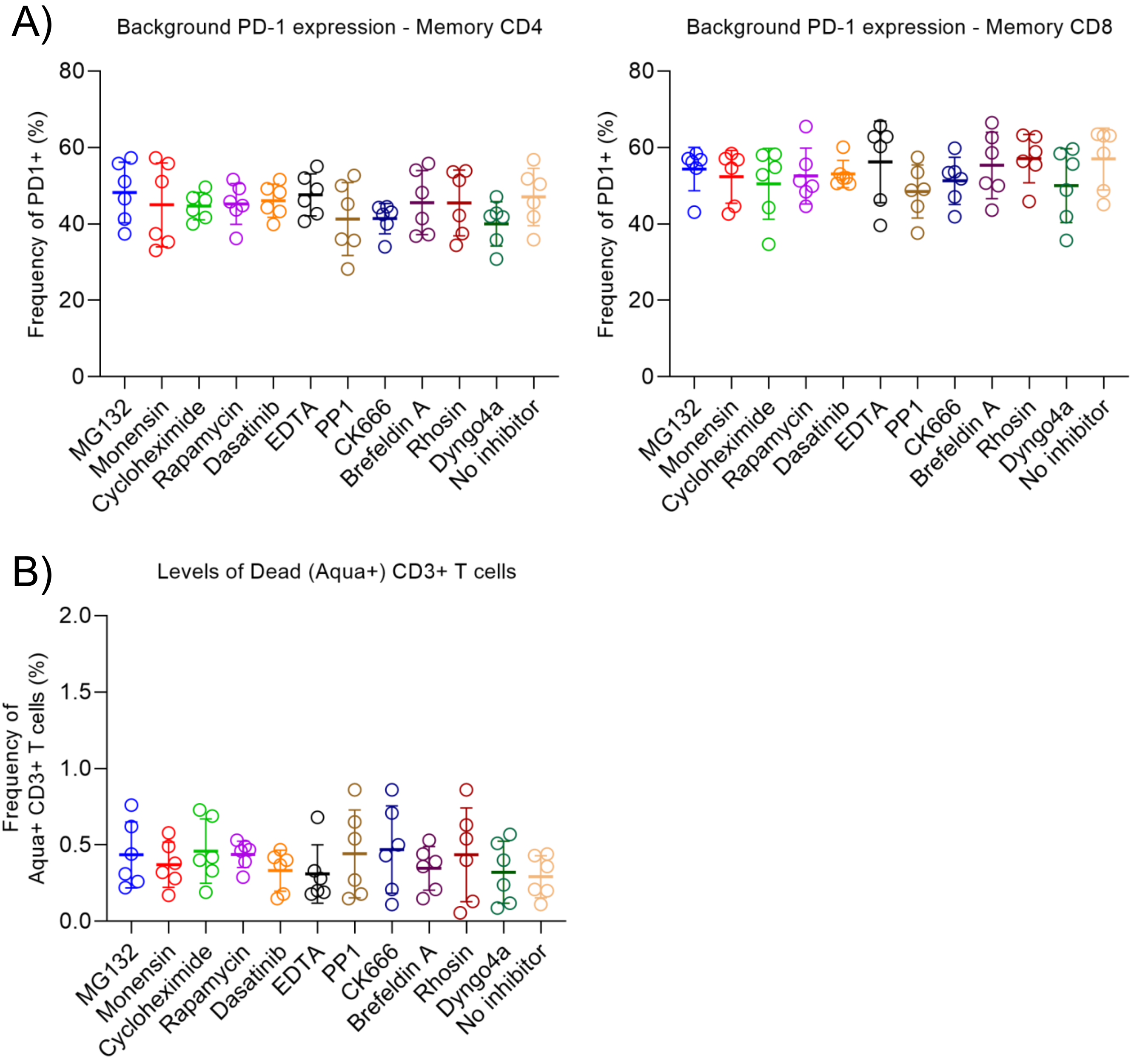

Supplementary Figure 4

Effect of inhibitors on T cells after 24 hours. (A) Frequencies of homeostatic PD-1 expression in memory CD4 and CD8 and **(B)** cell viability ($CD3^+$ $Aqua^+$) were measured after 24 hours incubation with different inhibitors and measured by a two-tailed unpaired *t* test with Welch's correction (n = 6, no significant differences found)

Supplementary Figure 5

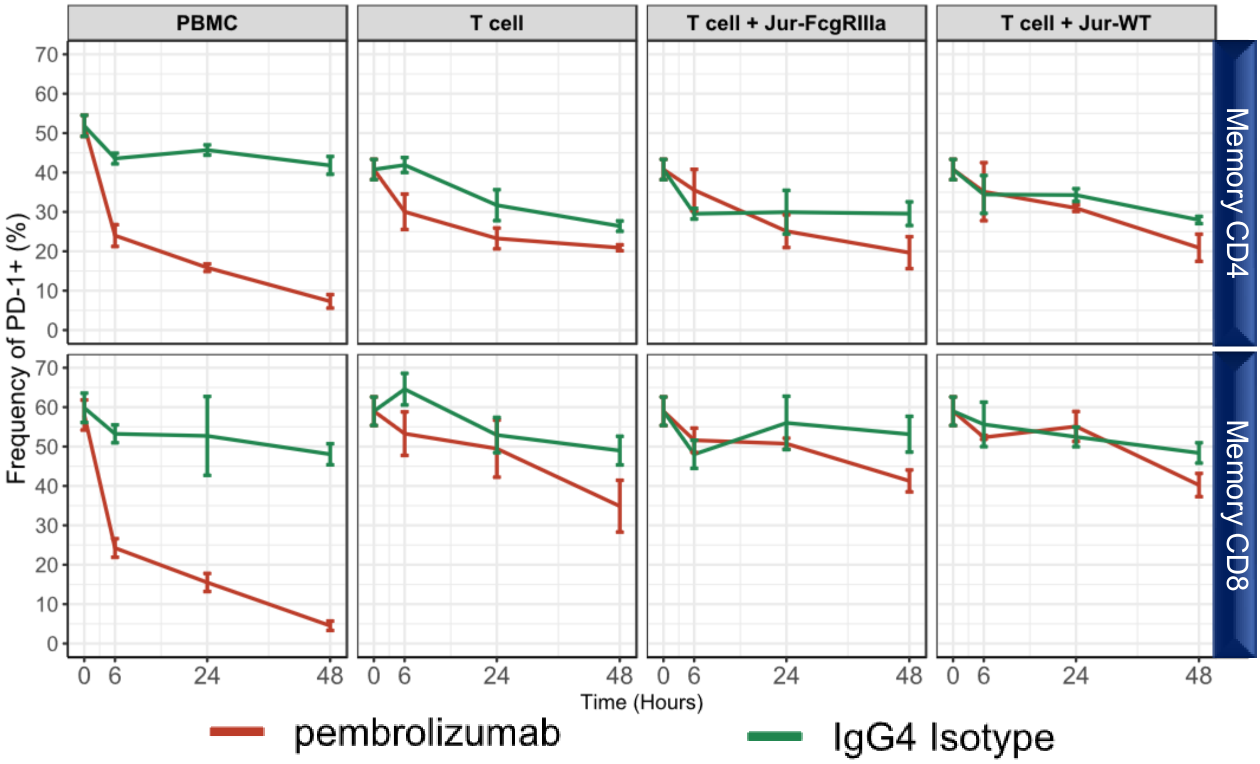

Supplementary Figure 5

Effect of CD16a on PD-1 downregulation. Total blood mononuclear cells (PBMC), purified T cells or purified T cells cocultured with WT Jurkats or Jurkats overexpressing FcγRIIIa/CD16a (5:1 ratio) were measured for PD-1 downregulation in the presence of pembrolizumab or IgG4 control. (n = 2 donors, averaged triplicates) Data represented as mean ± SD.

Supplementary Figure 6

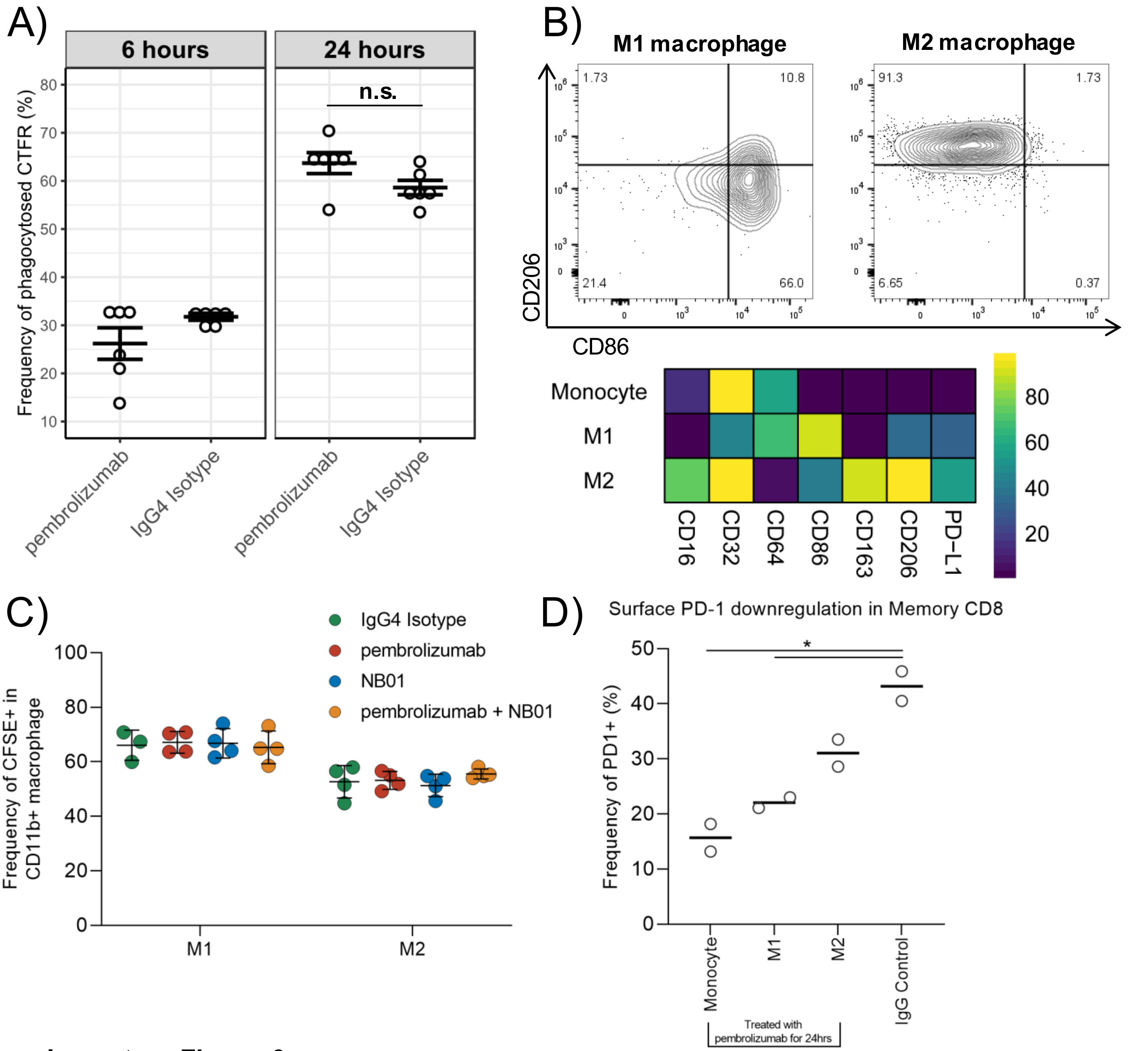

Supplementary Figure 6

ADCP activity and PD-1 downregulation in differentiated macrophages with PD-1 antibody treatment. (A) Phagocytic capacity measured in monocytes using purified T cells labeled with CTFR and cocultured with autologous monocytes incubated with pembrolizumab or IgG4 control for 24 hours (n= 6 donors). **(B)** Phenotype of M1 and M2 differentiated macrophages and expression heatmap of associated markers **(C)** Phagocytic capacity was measured in activated, M1-/M2-differentiated macrophages from healthy donors cocultured for 24 hours with autologous purified T cells labeled with CFSE. Data represented as mean ± SD and measured by a two-tailed unpaired *t* test with Welch's correction (n = 3-4 donors, no significant differences found). **(D)** Downregulation of PD-1 on autologous memory CD8 by pembrolizumab in coculture with monocytes, M1, or M2 differentiated macrophages after 24 hours compared to IgG4 control cocultured with monocytes. Data represented as mean (n = 2 donors) and measured by a two-tailed unpaired *t* test with Welch's correction; * denotes $p < 0.05$.
